# Centrally Administered Cortistation-14 Induces Antidepressant-Like Effects in Mice via Mediating Ghrelin and GABA_A_ Receptor Signaling Pathway

**DOI:** 10.3389/fphar.2018.00767

**Published:** 2018-07-19

**Authors:** JinHong Jiang, YaLi Peng, XueYa Liang, Shu Li, Xin Chang, LongFei Li, Min Chang

**Affiliations:** Key Laboratory of Preclinical Study for New Drugs of Gansu Province, School of Life Sciences, Institute of Biochemistry and Molecular Biology, Lanzhou University, Lanzhou, China

**Keywords:** cortistatin-14, antidepressant effects, somatostatin receptors (SSTRs), ghrelin receptor, GABA_A_ receptor, intranasal

## Abstract

Cortistatin-14 (CST-14), a recently discovered cyclic neuropeptide, can bind to all five cloned somatostatin receptors (SSTRs) and ghrelin receptor to exert its biological activities and co-exists with GABA within the cortex and hippocampus. However, the role of CST-14 in the control of depression processes is not still clarified. Here, we tested the behavioral effects of CST-14 in the in a variety of classical rodent models of depression [forced swimming test (FST), tail suspension test (TST) and novelty-suppressed feeding test]. In the models of depression, CST-14 produced antidepressant-like effects, and does not altered locomotor activity levels. And, we found that CST-14 mRNA and BDNF mRNA were significantly decreased in the hippocampus and cortex after mice exposed to stress. Further data show that i.c.v. administration of CST-14 produce rapid antidepressant effects, and does not altered locomotor activity levels. Then these antidepressant-like effects were significantly reversed by [D-Lys_3_]GHRP-6 (ghrelin receptor antagonist), but not c-SOM (SSTRs antagonist). Meanwhile, the effects of some neurotransmitter blockers indicates that only GABA_A_ system, but not CRF1 receptor, α/β-adrenergic receptor, is involved in the antidepressant effect of CST-14. The effects of the mTOR inhibitor (rapamycin), the PI3K inhibitor (LY294002) and the p-ERK1/2 inhibitor (U0126) suggesting that the ERK/mTOR or PI3K/Akt/mTOR signaling pathway is not involved in the antidepressant effects of CST-14. Interestingly, intranasal administration of CST-14 led to reducing depressive-like behavior, and near-infrared fluorescent experiments showed the real-time *in vivo* bio-distribution in brain after intranasal infusion of Cy7.5-CST-14. Taken all together, the results of present study point to a role for CST-14 in the modulation of depression processes via the ghrelin and GABA_A_ receptor, and suggest cortistation may represent a novel strategy for the treatment of depression disorders.

**Highlights:**

- CST-14 and BDNF mRNA are decreased in hippocampus and cortex once mice exposed to stress.
- i.c.v. or intranasal administration of CST-14 produce rapid antidepressant effects.
- NIR fluorescence imaging detected the brain uptake and distribution after intranasal CST-14.
- Antidepressant effects of CST-14 were only related to ghrelin and GABA_A_ system.
- Co-injection of CST-14 and NPS produce antidepressant effect, and do not impair memory.

## Introduction

Depression is a commonly occurring, life-threatening, and debilitating psychiatric disorder that is projected to become the second biggest contributor to the global burden of disease and disability for the year 2020, with the main symptoms of being feeling sad, anxious, hopeless or restless, and losing interest in activities (Binder and Nemeroff, 2010; Suzuki et al., 2015; Shaw et al., 2016; Tanriverdi et al., 2016; Laman-Maharg et al., 2018). Although numerous monoaminergic-based antidepressants such as tricyclic antidepressants (TCAs) and serotonin-norepinephrine reuptake inhibitors (SNRIs) are available, it takes a few weeks to several months for most patients to respond to these treatments (Carlier et al., 1998; Bailey et al., 2016; Nonen et al., 2016; Podkowa et al., 2016). Due to the various pathogenic factors involved in depression, many of the drugs fail to get remarkable effects and many patients are resistant to the treatment (Crisafulli et al., 2011; Bailey et al., 2016; Inoue et al., 2016; Roseman et al., 2017). Therefore, it is necessary to find new antidepressant drugs.

Cortistatin (CST), a novel neuroendocrine peptide with a cyclic configuration, was named after its abundantly cortical expression and inhibition the function, which is widely distributed in the central nervous system, endocrine system, and immune system, and is extremely conserved across multi-species (Mendezdiaz, 2004; de Lecea, 2008). An increasing number of articles were published during the past decade showing CST-14 moderates a broad range of physiological and pathological processes, including reduction of locomotor activity (de Lecea, 2008; Semenova et al., 2010), the production or formation of tumors (Cassoni et al., 2002), anti-inflammatory effect (Gonzalez-Rey et al., 2006; Gruber et al., 2014; Morell et al., 2014; Zhang et al., 2015a), induction of slow-wave sleep (Mendezdiaz, 2004; Gonzalez-Rey et al., 2015) and impairment of neuronal activity (Tallent et al., 2005) and inhibition of cell proliferation (Zhang et al., 2015b).

Cortistatin exhibits several biologically active forms, including CST-29, CST-17, and CST-14. CST-14 shares high structural homology with somatostain-14 (Spier and de Lecea, 2000; Deghenghi et al., 2001b; Kumar, 2005; Gahete et al., 2010; Fani et al., 2012; Takeuchi et al., 2016), and binds to all five cloned somatostatin receptors (SSTRs) (Spier and de Lecea, 2000; Kumar, 2005). Besides, CST-14 can also bind to ghrelin receptor to exert its biological activities (Deghenghi et al., 2001b; Carlini et al., 2012; Aourz et al., 2014). Recent work has begun to draw connections between ghrelin system and depression (Carlini et al., 2012; Poretti et al., 2015). Ghrelin, as a new endogenous antidepressant, must have its benefits, the mechanisms of ghrelin’s antidepressant-like action have become the most interested topics for researchers (Poretti et al., 2015). Studies demonstrated that ghrelin may play a major role in hippocampal neurogenesis in the treatment of depression (Poretti et al., 2015). Moreover, depression has been associated with alterations of growth factors, particularly in brain-derived neurotrophic factor (BDNF). In this experiment, BDNF signaling molecules were used as positive controls (Borsoi et al., 2015; Lepack et al., 2016; Ludka et al., 2016; Rosa et al., 2016). The localization of the SSTRs and ghrelin receptor in brain has prompted speculation as to a potential role of CST-14 in the control of depression processes. To investigate this possibility, a primary goal of this study was to determine whether central injection of CST-14 could produce a substantial impact on antidepressant-related behavior in animal models through the forced swimming test (FST), tail suspension test (TST), novelty suppressed feeding test (NSF), and open field test (OF), which are commonly employed as the animal models to assess antidepressants (Porsolt et al., 1977; Steru et al., 1985). Second goal of this study was to determine the underlying mechanism of CST-14 on antidepressant-like behavior. Additionally, previous research from our laboratory indicates that intrahippocampal injection of CST-14 impairs cognition memory consolidation in mice (Jiang et al., 2017). Due to the memory-enhancing effects of Neuropeptide S (NPS) in mice novel object and object location recognition tasks (Han et al., 2013), we also evaluated the effect of joint administration of CST-14 and NPS on the antidepressant-like action in the FST, and learning and memory in the novel object recognition task (NOR).

Intranasal (IN) infusion allows peptides to rapidly enter the CNS via intracellular neuronal olfactory and extracellular trigeminal associated pathways, bypassing the blood brain barrier to effectively interact with their receptors in multiple brain regions. Thus, our work also underscores the clinical potential of intranasal CST-14 treatment to regulate antidepressant effect.

## Materials and Methods

### Animals

Male mice (Kunming strain of Swiss), weighing 23–25 g at the beginning of the experiments, were obtained from the Experimental Animal Center of Lanzhou University, China. Animals were housed in cages (sizes 20 × 30, humidity 45–50%, the bedding-wood shavings, 8 animals/cage) in an animal room maintained at 22 ± 2°C and on a 12 h light-dark cycle (8 A.M.–8 P.M.) with free access to tap water and food. All the protocols in this study were approved by the Ethics Committee of Lanzhou University, China (permit number: SYXK Gan 2009–0005).

### Surgery

Surgical implantation of cannula into the lateral ventricle was conducted as described in our previous report (Patel et al., 2012; Jiang et al., 2015b). Each mouse (20–24 g) was anesthetized with sodium pentobarbital (Sigma-Aldrich Co., United States, at a dose of 70 mg/kg) and placed in a stereotaxic frame (Leica, Germany). Using the atlas of Paxinos et al. (2001), 8 mm 26-gauge stainless-steel guide cannulas, closed by stylets, were implanted over the lateral ventricle (0.5 mm posterior to bregma, 1.0 mm lateral to midline, 2.0 mm ventral to skull surface). After surgery, mice were housed individually and allowed to recover for 5–7 days. All experiments were carried out between 9:00 A.M. and 6:00 P.M.

At the end of the experiments, the correct position and the permeability of the cannula were checked by injecting methylene blue into the cannula. Following the behavioral studies, each mouse was sacrificed by cervical dislocation, brains were then dissected, and the position of each cannula was determined. Data exhibiting the diffusion of methylene blue in the ventricles was analyzed. All mice were used only once.

### Drugs

Cortistatin-14, c-SOM, and [D-Lys_3_]GHRP-6 were synthesized by a standard Fmoc-based solid-phase synthetic method, and the obtained crude peptides were purified to homogeneity with preparative HPLC. The purity of peptides was ascertained by analytical HPLC, and the structure assignment was performed by ESI-TOF MS. The purity and structure of CST-14 is described in our previous report (Jiang et al., 2017). Purified CST-14 was dissolved in artificial CSF (vehicle) containing (in mM) 126.6 NaCl, 27.4 NaHCO_3_, 2.4 KCl, 0.5 KH_2_PO_4_, 0.89 CaCl_2_, 0.8 MgCl_2_, 0.48 Na_2_HPO_4_, and 7.1 glucose, pH 7.4. Picrotoxin, antalarmin, phentolamine (PHE, i.p., 1 mg/kg), propranolol hydrochloride (i.p., 1 mg/kg), U0126 (i.c.v., 5 μg/mouse), LY294002 (i.c.v., 10 nmol/mouse) and Rapamycin (i.c.v., 0.2 nmol/mouse) were bought from Sigma-Aldrich (United States), and these compounds were dissolved in aCSF. Picrotoxin (PTX, i.p., 1 mg/kg) was dissolved in 1% dimethylsulfoxide for the *in vivo* study. Antalarmin (i.c.v., 25 μg/mouse), purchased from Tocris Bioscience (Bristol, United Kingdom), was dissolved in cremaphor/ethanol at 2:1 to a concentration of 30 mg/ml and kept at 4°C and was diluted in saline immediately before injected.

Different doses of CST-14 or vehicle were infused over a period of 2 min via two 5 μL Hamilton syringes mounted on a Microdrive pump (KD Scientific). All drugs and vehicle were injected using a 32-gauge stainless steel injector placed in and projecting 0.5 mm below the tip of the cannula. Infusion cannula remained in place for 1 min after infusion to allow for drug diffusion. Ten minutes after CST-14 administration, the mouse was subjected to behavioral testing. In addition, the selection of the doses was based on previous reports and our preliminary observations (Aourz et al., 2014; Jiang et al., 2017).

### Forced Swimming Test (FST)

The procedures for the forced swimming test were reported by Porsolt et al. (1977) and Rebai et al. (2017). Briefly, each mouse was tested in a glass cylinder (height 25 cm, diameter 15 cm) filled with water (height 12 cm, temperature 24 ± 1°C). The immobility time was measured during the last 4 min of the 6 min testing period. Mice were randomly assigned to experimental groups.

### Tail Suspension Test (TST)

The TST was carried out according to the procedure of Steru et al. (1985). Briefly, each mouse, positioned horizontally 75 cm above the tabletop, were individually suspended on the rod by adhesive tape placed approximately 1 cm from the tip of the tail. They were allowed to hang, and the immobility duration was recorded for the last 4 min of the 6 min testing period. The testing room was kept quiet during the entire test.

### Novelty-Suppressed Feeding Test (NSFT)

Mice were singly housed and food-deprived for 24 h in freshly made home-cages. Two normal chow diet pellets were placed on an inverted weighingboat platform (10 cm × 10 cm × 1.5 cm) in the center of an open-field arena (40 cm × 40 cm). Ten minutes after drug administration, mice were introduced into a corner of the arena. The time needed for the mice to take a bite of food was recorded over a 10 min period by a trained observer. After the test, the mice were returned to their home cage containing pre-weighed food pellets, and latency to start biting the pellet, as well as consumption was recorded for a period of 10 min. There was no significant change in home cage latency (data not shown) (Liu et al., 2017).

### Locomotor Activity Tests

The open field, a square arena (50 cm × 50 cm × 40 cm^3^) with a black floor and Plexiglas walls, was illuminated by overhead fluorescent lighting (120 lx) and employed to examine the locomotor activity of mice. Training was conducted as previously reported (Jiang et al., 2015a). Mice were placed in the center of the open field and left to freely explore for a 30 min. Locomotor activity including distance traveled and rearing counts was measured by open field test analysis system (TME, Chengdu, China).

### Object Recognition Task (NOR)

The procedure of NOR task was based on our previous report (Jiang et al., 2015c), and that described by Maroun and Akirav (2009) and Okamura et al. (2011). Briefly, each mouse was tested in its home cage in a sound-attenuated room with somber lighting. The general procedure consisted of a training phase and a retention phase, separated by a delay. Each mouse was handled 3 min per day for 3 consecutive days prior to training. During the training phase, two identical objects were placed in the opposite sides of the home cage. The sample phase ended when mouse had explored two identical objects for a total of 20 s. Mouse showing a total exploratory time (TET) <20 s within 10 min was excluded. In the test phase, a familiar object from the training phase and a novel object were placed in the same locations as in the training phase. The test phase was ended when mouse had explored two objects for a total of 25 s, or after 5 min had passed, which ever came first. All objects were made of plastic or glass, similar in size (4–5 cm high) but different in color and shape. There were several copies of each object for use interchangeably. Throughout the experiments, the objects used as novel or familiar were counterbalanced to exclude possible preference for special object. Moreover, the locations of the novel and familiar objects were also counterbalanced in the test to exclude possible spatial bias. Objects were cleaned thoroughly between trials to ensure absence of olfactory cue. Exploration was defined as sniffing or touching object with nose and/or forepaws. Resting against or turning around object was not considered exploratory behavior. The time spent exploring each object was recorded by an observer blind to the treatments. A discrimination index (DI) in the test phase was calculated as a percentage of the time spent exploring the novel object over the total time spent exploring both objects. A DI of 50% corresponds to the chance level and a higher DI reflects intact recognition memory.

### Quantitative PCR

Real-time RT-PCR was performed according to the instructions and report (Liu et al., 2014). Total RNA was extracted using Trizol reagent (TaKaRa) and 1 μg of RNA in each sample was reversely transcribed into a single-stranded complementary DNA with the 5X PrimeScript RT Master Mix (TaKaRa) following manufacturer’s instructions. Amplification was carried out in a 25 μL reaction mixture consisting of 12.5 μL of 2X SYBR Premis Ex TaqTM II, 2 μL of cDNA, 1 μL of forward primer, 1 μL of reverse primer and 8.5 μL of ddH_2_O; and was run under the following conditions: 95°C for 30 s, followed by 40 cycles of 95°C for 5 s, 58°C for 30 s and 72°C for 30 s. The primer pair P1/P2 was used in the RT-PCR assay to identify the expression level of CST-14 and BDNF gene in the hippocampus and cortex of control and depression groups. The primers used in quantitative real-time PCR were CST-14 (forward 5′-GGAGCGGCCTTCTGACTTTCC-3′ and reverse 5′-GCCTTTCCTGGCTCTTGGACA-3′), and BDNF (forward 5′-AACCATAAGGACGCGGACTTGT-3′ and reverse 5′-ATGTTTGCGGCATCCAGGTAAT-3′) GAPDH (forward 5′-GCCACAGACGTCACTTTCCTAC-3′ and reverse 5′-CGGGAACACAGTCACATACCA-3′). The GAPDH was used as an endogenous control in each sample.

### NIR Fluorescence Imaging

To confirm the penetration of the CST-14 in the brain tissue of mice, the fluorescent dye Cy7.5 was used. The specific synthesis method is shown in **Figure 8B**. NIR fluorescence imaging was performed using a whole-mouse imaging system (Imaging Station IVIS Lumina II, Caliper). Mice were injected intranasally with Cy7.5-CST-14 and Cy7.5 alone into nasal cavity after 30 min. Mice were sacrificed and perfused with 20 mL of saline for removal of residual blood in the brain. The whole perfused brains were removed and placed into an imaging system. Images were captured by the CCD camera embedded in the imaging system and analyzed using the Lumina II Living Imaging 4 software.

### Statistical Analysis

**FIGURE 1:**
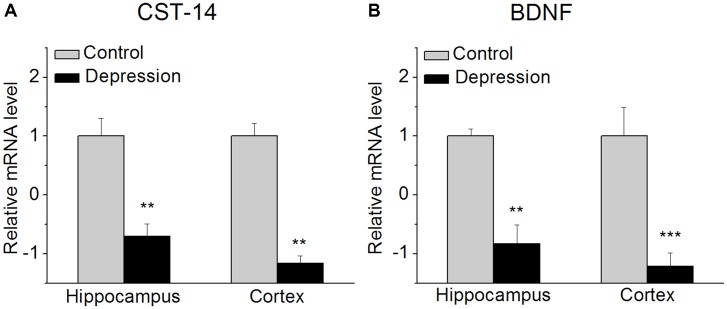
CST-14 and BDNF mRNA level in the hippocampus and cortex. **(A)** The expression level of CST-14 mRNA. **(B)** The expression level of BDNF mRNA. The results revealed that these are significantly decreased in the expression of CST-14 mRNA and BDNF mRNA in the hippocampus and cortex of mice experienced forced swimming test than control. All data are expressed as mean + SEM and were analyzed by one-way ANOVA followed by Dunnett’s *post hoc* test. *N* = 6/group. ^∗∗^*p* < 0.01 and ^∗∗∗^*p* < 0.001 compared with control.

**FIGURE 2:**
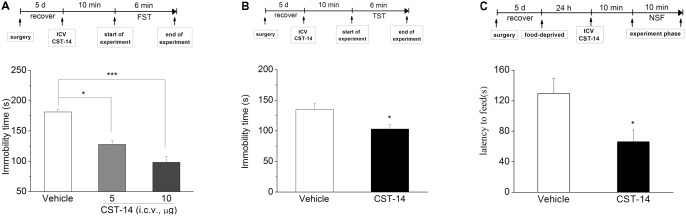
The antidepressant effects of CST-14 in the FST, TST, and NSFT of mice. Schematic representation of the experimental schedule. **(A)** i.c.v. administration of CST-14 (1 and 5 μg), 10 min before the FST, dose-dependently produce rapid antidepressant effects. **(B)** The antidepressant-like effect of CST-14 (5 μg) in the TST in mice. **(C)** The antidepressant-like effect of CST-14 (5 μg) in the NSFT in mice. All data are expressed as mean + SEM and were analyzed by two-way ANOVA followed by Dunnett’s *post hoc* test. *N* = 8–11/group. ^∗^*p* < 0.0 and ^∗∗∗^*p* < 0.001 compared with control.

**FIGURE 3:**
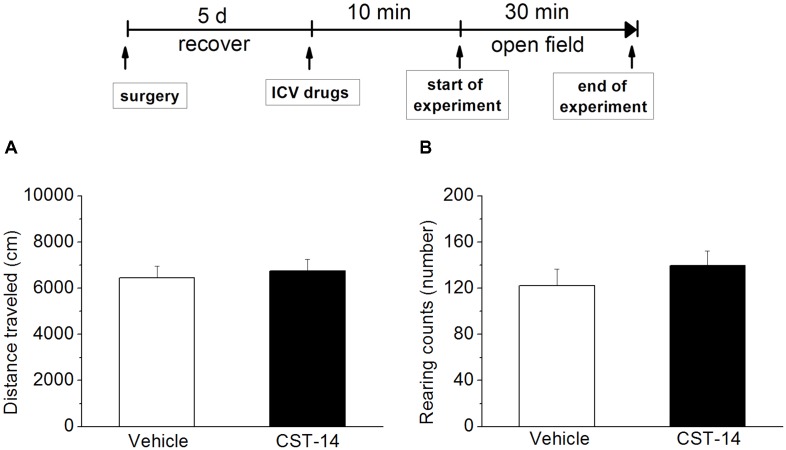
Effects of CST-14 (5 μg; i.c.v.) on the locomotor behavior in mice in the open field. **(A,B)** displays the effect exerted by CST-14 on distance traveled **(A)** and rearing counts **(B)** over the 30 min observation period. All data are expressed as mean + SEM and were analyzed by one-way ANOVA followed by Dunnett’s *post hoc* test. *N* = 8/group.

**FIGURE 4:**
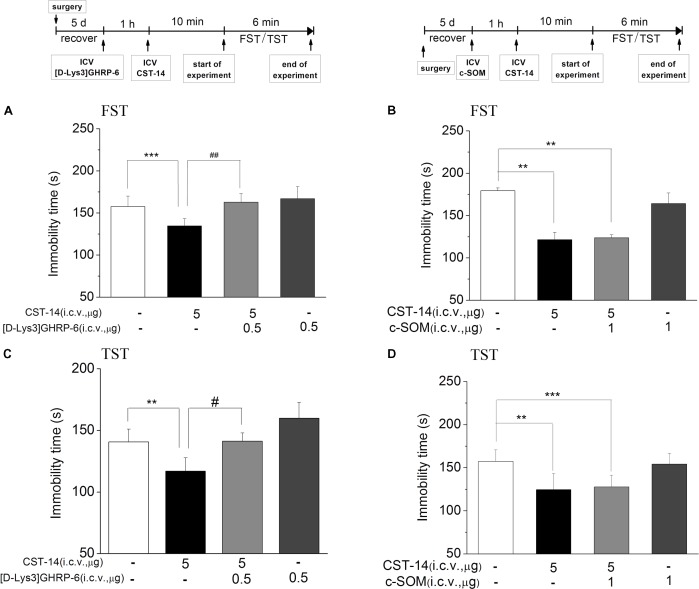
The effects of [D-Lys3]GHRP-6 **(A)** and c-SOM **(B)** on the antidepressant-like effect induced by CST-14 in the FST and TST of mice. Schematic representation of the experimental schedule. i.c.v. infusion of [D-Lys3]GHRP-6 (0.5 μg), 60 min prior to CST-14 administration (5 μg; i.c.v.), was significantly blocked antidepressant effects induced by CST-14 in FST **(A)** and TST **(C)**. Infusion of c-SOM (1 μg; i.c.v.), 60 min prior to CST-14 administration (5 μg; i.c.v.), was not antagonized the antidepressant effects of CST-14 in FST **(B)** and TST **(D)**. All data are expressed as mean + SEM and were analyzed by two-way ANOVA followed by Dunnett’s *post hoc* test. *N* = 10–12/group. ^∗∗^*p* < 0.01 and ^∗∗∗^*p* < 0.001 compared with control; ^#^*p* < 0.05 and ^##^*p* < 0.01 compared with CST-14 + vehicle group.

**FIGURE 5:**
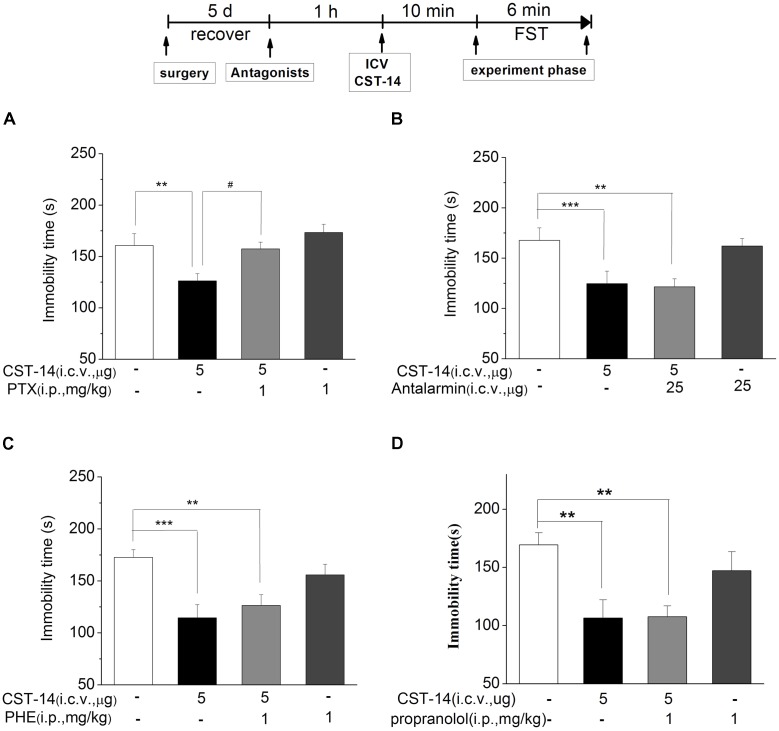
The effects of PTX, antalarmin, PHE and propranolol on the antidepressant-like effects of CST-14 in the forced swimming test of mice. Schematic representation of the experimental schedule. **(A)** Injection of PTX (1 mg/kg; i.p.), 60 min prior to CST-14 administration (5 μg; i.c.v.), was significantly blocked antidepressant effects induced by CST-14 in FST. **(B–D)** i.c.v. infusion of antalarmin (25 μg; i.c.v.), or PHE (1 mg/kg; i.p.), or propranolol (1 mg/kg; i.p.), 60 min prior to CST-14 administration (5 μg; i.c.v.), was not blocked antidepressant effects induced by CST-14 in FST. All data are expressed as mean + SEM and were analyzed by two-way ANOVA followed by Dunnett’s *post hoc* test. *N* = 10–12/group. ^∗∗^*p* < 0.01 and ^∗∗∗^*p* < 0.001 compared with control; ^#^*p* < 0.05 compared with CST-14 + vehicle group.

**FIGURE 6:**
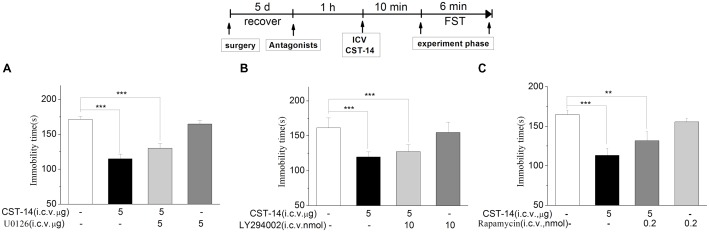
The effects of U0126, LY294002, rapamycin on the antidepressant-like effects of CST-14 in the forced swimming test of mice. Schematic representation of the experimental schedule. **(A)** Injection of U0126 (5 nmol/mouse, i.c.v.), 30 min prior to CST-14 administration (5 μg; i.c.v.), could not blocked antidepressant effects induced by CST-14 in FST. **(B,C)** i.c.v. infusion of LY294002 (10 nmol/mouse, i.c.v.), or rapamycin (0.2 nmol/mouse, i.c.v.), 60 min prior to CST-14 administration (5 μg; i.c.v.), was not blocked antidepressant effects induced by CST-14 in FST. All data are expressed as mean + SEM and were analyzed by two-way ANOVA followed by Dunnett’s *post hoc* test. *N* = 9–11/group. ^∗∗^*p* < 0.01 and ^∗∗∗^*p* < 0.001 compared with control.

**FIGURE 7:**
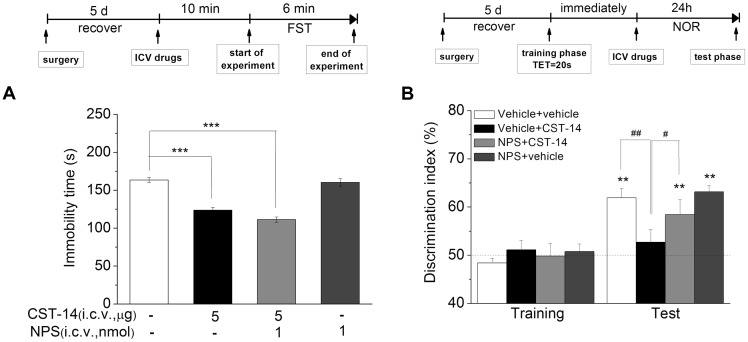
**(A)** The antidepressant-like effect of the joint administration of CST-14 (5 μg; i.c.v.) and NPS (1 nmol; i.c.v.) in the FST of mice. **(B)** The effect of co-administration of CST-14 (5 μg; i.c.v.) and NPS (1 nmol; i.c.v.) on the object recognition memory task of mice. Schematic representation of the experimental schedule. All data are expressed as mean + SEM and were analyzed by one-way ANOVA followed by Dunnett’s *post hoc* test. *N* = 10–11/group. ^∗∗^*p* < 0.01 and ^∗∗∗^*p* < 0.001 compared with control; ^#^*p* < 0.05 and ^##^*p* < 0.01 compared with CST-14 + vehicle group.

Data are presented as means ±± SEM. Statistical analyses were conducted using SPSS 18.0. Among **Figures 4–7** were assessed by two-way ANOVA, followed by Dunnett’s *post hoc* test for multiple comparisons. Among **Figures 1–3** were assessed by one-way ANOVA, and *p* < 0.05 was considered as significance.

## Results

### The Expression of CST-14 and BDNF mRNA in the Hippocampus and Cortex in Mice

We investigated the expression of CST-14 mRNA in the hippocampus and cortex of mice immediately after experiencing forced swimming test. The results revealed that these are significantly decreased in the expression of CST-14 mRNA than control, while BDNF mRNA, as a positive control, is also significantly decreased (**Figure 1**).

### Antidepressant-Like Effects of CST-14 in the FST, TST, NSFT, and Open Field

In the FST, i.c.v. injection of CST-14 (5 μg) induced a dose-related reduction in immobility time [*F*(2,24) = 33.227, *P* < 0.001] (**Figure 2A**), when compared with control group. Meanwhile, as demonstrated in **Figure 2B**, the immobility time was shortened by i.c.v. administration of CST-14 [*F*(1,15) = 5.625, *P* < 0.05] in the TST, when compared with control group. The NSFT assay showed that CST-14 significantly shortened the latency to begin feeding, suggesting that CST-14 led to reducing depressive-like behavior [*F*(1,16) = 4.713, *P* < 0.05].

Additionally, in order to rule out the possibility that CST-14 had increased immobility time by a non-specific reduction in general activity, the effect of CST-14 on locomotor activity was examined by open field test. The results showed that there was no significant difference between CST-14 (5 μg/mouse) and control groups in locomotor activity (*P* > 0.05 at distance traveled and rearing counts) (**Figure 3**).

### The Underlying Mechanisms of Antidepressant Effects of CST-14 in the FST

#### Effects of Somatostatin Receptor_1-5_ and Ghrelin Receptor Antagonists on the Antidepressant-Like Effect Induced by CST-14

c-SOM, an antagonist of the somatostatin receptor_1-5_, did not lead to a significantly inhibition of the antidepressant-like effect induced by CST-14 in the FST (*P* > 0.05) (**Figure 4B**). Then in present experiment, pretreatment with c-SOM (1 μg, i.c.v.), 60 min prior to CST-14 (5 μg; i.c.v.), was not significantly blocked the antidepressant-like effects induced by CST-14 in the FST (*P* > 0.05 for c-SOM+CST-14 vs. vehicle+CST-14, **Figures 4B,D**).

Meanwhile, pretreatment with [D-Lys_3_]GHRP-6 (0.5 μg, i.c.v., an antagonist of the ghrelin receptor), 60 min prior to CST-14 (5 μg; i.c.v.), could fully block the antidepressant-like behaviors of CST-14 (**Figure 4A**) [*F*(3,32) = 7.141, *R*^2^ = 0.461, *p* < 0.01 for [D-Lys_3_]GHRP-6+CST-14 vs. vehicle+CST-14] and (4C) [*F*(3,31) = 4.18, R^2^ = 0.337, *p* < 0.05 for [D-Lys_3_]GHRP-6+CST-14 vs. vehicle+CST-14]. These data indicate that the antidepressant-like effect of CST-14 is involved in the ghrelin receptor system, but not somatostatin receptor system.

#### Effects of GABA_A_ Receptor, CRF1 Receptor, and α/β-Adrenaline Receptor on the Antidepressant-Like Effect Induced by CST-14

We investigated the effects of PTX, an antagonist of the GABA_A_ receptor, on the antidepressant-like effect induced by CST-14 in the FST of mice. The results revealed that PTX could block the antidepressant-like effect of CST-14 [*F*(3,33) = 5.58, *R*^2^ = 0.341, *p* < 0.05 for PTX+CST-14 vs. vehicle+CST-14, **Figure 5**]. Meanwhile, PTX itself had no effects on these tests (**Figure 5A**).

The results revealed that antalarmin could not block the antidepressant-like behaviors of CST-14 (*P* > 0.05 for antalarmin+CST-14 vs. vehicle+CST-14, **Figure 5B**). Then we investigated the effects of phentolamine (PHE, an antagonist of the α-adrenaline receptor) and propranolol (an antagonist of the β-adrenaline receptor) on the antidepressant-like effects of CST-14 in the FST. The results indicated that CST-14 still elicited the antidepressant-like effects in the presence of PHE (*P* >> 0.05 for PHE+CST-14 vs. vehicle+CST-14, **Figure 5C**) and propranolol (*P* > 0.05 for propranolol+CST-14 vs. vehicle+CST-14, **Figure 5D**) in the FST. These data suggest that the antidepressant-like effect of CST-14 is not involved in the CRF1 receptor or α/β-adrenaline receptor system.

#### Effects of p-ERK Inhibitor, mTOR Inhibitor or p-PI3K/AKT Inhibitor on the Antidepressant-Like Effect Induced by CST-14

An increasing number of human diseases have been linked to dysregulation of mTOR, including depression. **Figure 6** shows the effect of inhibition of the p-ERK by U0126 (5 nmol/mouse, i.c.v.), the upstream Akt activator PI3K by LY294002 (10 nmol/mouse, i.c.v.), p-mTOR by rapamycin (0.2 nmol/mouse, i.c.v.) in the antidepressant-like effect of CST-14 in the FST. The two-way ANOVA revealed that there are no significant differences for U0126 treatment [*F*(3,31) = 1.51, *R*^2^ = 0.148, *p* > 0.05 for U0126+CST-14 vs. vehicle+CST-14, **Figure 5**], LY294002 treatment [*F*(3,30) = 2.173, *R*^2^ = 0.179, *p* > 0.05 for LY294002+CST-14 vs. vehicle + CST-14, **Figure 5**] and rapamycin treatment [*F*(3,31) = 1.612, *R*^2^ = 0.151, *p* > 0.05 for rapamycin+CST-14 vs. vehicle+CST-14, **Figure 5**]. These data showing that the antidepressant-like effect of CST-14 was not prevented by the administration of p-ERK inhibitor, PI3K inhibitor and p-mTOR inhibitor to mice.

#### The Effect of Joint Administration of CST-14 and NPS on the Depression and Memory

Although i.c.v. administration of CST-14 produced an antidepressant action, it can impair the memory system at the same dose as well. NPS was reported to enhance memory and mitigate memory impairment induced by MK801, scopolamine or Aβ_1–42_ in mice (Okamura et al., 2011; Han et al., 2013). Thus, we investigated the effect of joint administration of CST-14 and NPS on the depressant-like action in the FST, and learning and memory in the NOR. Two-way ANOVA revealed that i.c.v. administration of CST-14 (5 μg) when given in a combination with NPS (1 nmol) did not impair the object recognition memory [*F*(3,31) = 5.31, *P* < 0.05] (**Figure 7B**), and significantly shortened the immobility time of mice in the FST [*F*(3,33) = 57.467, *P* < 0.001] (**Figure 7A**).

#### Intranasal Administration of CST-14 Evoked the Antidepressant-Like Effects in the FST

**FIGURE 8:**
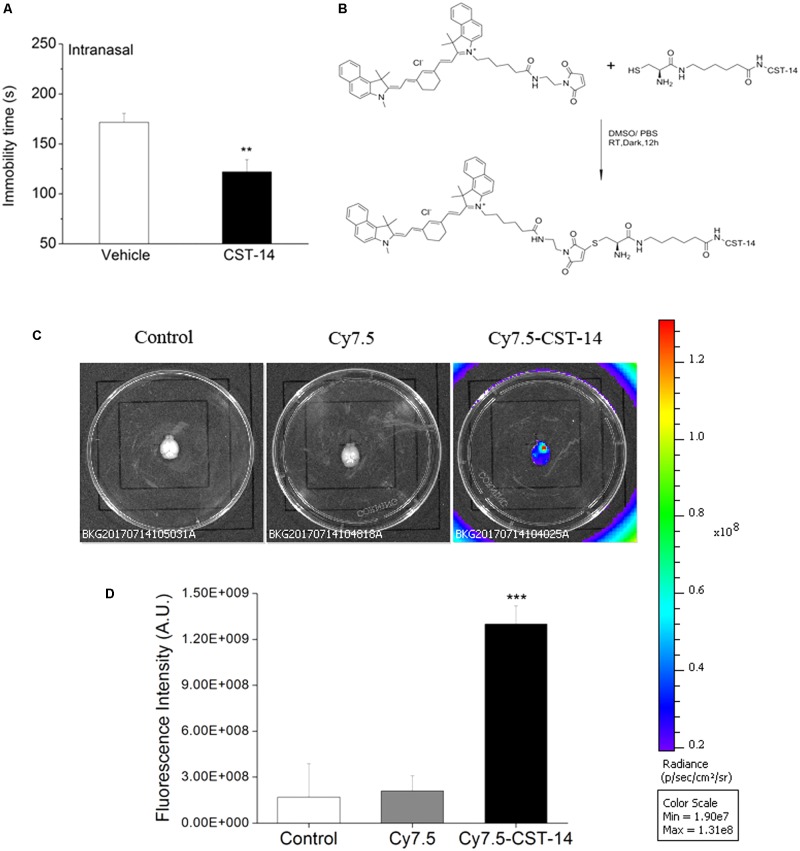
**(A)** Intranasal administration of CST-14 (50 μg), 2 h before the FST, produce rapid antidepressant effects. **(B)** The synthesis method and structure of Cy7.5-CST-14. **(C)** *Ex vivo* imaging of perfused mouse brains 30 min after intranasal injection of the Cy7.5-CST-14, Cy7.5 alone and saline as controls. **(D)** Fluorescence intensity after intranasal injection of Cy7.5-CST-14, Cy7.5 alone and saline. All data are expressed as mean + SEM and were analyzed by one-way ANOVA followed by Dunnett’s *post hoc* test. *N* = 3/group. ^∗∗^*p* < 0.01 and ^∗∗∗^*p* < 0.001 compared with control.

When intranasal administration of CST-14 (50 μg) exerted an acute antidepressant-like effect, the immobility time (CST-14: 122.14 ± 12.19, control: 171.6 ± 8.99) was significantly shortened. There was significant difference between CST-14 and control groups in immobility time (**Figure 8A**).

To demonstrate the delivery of CST-14 into the mouse brain, *in vivo* fluorescence imaging experiments was performed. Mice were killed and perfused for removal of the residual blood in brain, and the brains were dissected for *ex vivo* fluorescence imaging. Cy7.5-CST-14 distributed of the whole brain immediately at 30 min after intranasal injected (**Figure 8C**). **Figure 8D** shows the real-time *in vivo* bio-distribution for Cy7.5-CST-14. Brains from control mice treated with Cy7.5 or saline alone displayed no significant signal.

## Discussion

The present data for the first time provide evidence of i.c.v. administration of CST-14, a novel neuropeptide, produces rapid antidepressant effects in the FST, TST, NSFT. The antidepressant effects induced by CST-14 were mediated by the activation of ghrelin receptor, but not somatostatin system. In detail, i.c.v. injection of CST-14 (5 and 10 μg/mouse) significantly shortened immobility time at a dose-dependent manner compared to the control group in FST, TST, NSFT. Drugs enhancing locomotor activity evoke a “false” positive effect in these FST (Reichert et al., 2015). To investigate this possibility, we also evaluated the effect of CST-14 on locomotor activity. Our data showed that i.c.v. administration of CST-14 had no significant differences in the locomotor activity test, which provided the direct evidence that centrally infused CST-14 produced antidepressant effects in mice.

Given the structural similarities between CST-14 and SRIF, CST-14 binds with nanomolar affinity to each of the five cloned SSTRs, which were proposed as potential target sites for the biological effects of CST-14 (Spier and de Lecea, 2000; Markovics et al., 2012; Aourz et al., 2014). Thus, we used c-SOM, a SSTRs antagonist, to research the effects of it in the antidepressant effects induced by CST-14. The results show that the antidepressant effects of CST-14 could not be blocked by c-SOM, suggesting that the somatostatin system does not participate in the antidepressant effects of CST-14. In addition, Deghenghi et. al reported that the ghrelin receptor has been proposed as a target for the biological effects of CST-14 (Deghenghi et al., 2001a,b). Recent work has begun to draw connections between ghrelin system and depression, and the mechanisms of ghrelin as a new endogenous antidepressant-like action have become the most interesting topics for researchers (Poretti et al., 2015). Due to an important role of ghrelin system in depression process, our results, for the first time, demonstrated that [D-Lys_3_]GHRP-6, a selective ghrelin receptor antagonist (Patel et al., 2012), was able to block the antidepressant effects of CST-14 in the FST and TST of mice. These current data suggest that ghrelin receptor, but not somatostatin system, is involved in the actions of CST-14 in depression process.

Further experiments demonstrated that PTX, a GABA_A_ receptor antagonist (Das et al., 2003), were able to obstruct the antidepressant effects of CST-14 in the FST of mice, indicating that GABA system is essential for the antidepressant-like actions of CST-14 (Li et al., 2016). The GABA hypothesis of depression has found increasing support that α2/α3 GABA_A_ receptor modulators, the primary substrates for many psychotropic agents, may serve as novel antidepressants (Das et al., 2003; Mohler, 2012; Pytka et al., 2016). Juhee et al and Cruz et al demonstrated the interaction of ghrelin and GABAergic neurons in rat central nervous system (Cruz et al., 2013; Haam et al., 2014). Previous research from our laboratory indicates that ghrelin receptor and GABA system are involved in the actions of CST-14 in memory process (Jiang et al., 2017). Meanwhile, cortistatin is expressed in GABAergic neurons of the cerebral cortex and the hippocampus (Sanchez-Alavez et al., 2000; Mendez-Diaz et al., 2005; Tallent et al., 2005).

Besides, dysregulation of the hypothalamic-pituitary-adrenal (HPA) axis has been observed in major depression, and normalization of HPA axis has been suggested to play a role in the mechanisms of action of antidepressants (Swiergiel et al., 2008; Chen and Williams, 2012; Crowley and Girdler, 2014). Therefore, we also investigated the role of antalarmin (an antagonist of the CRF1 receptor), PHE (an antagonist of the α-adrenergic receptor) and propranolol (an antagonist of the β-adrenergic receptor) on the antidepressant effects of CST-14. The results demonstrated that antalarmin, PHE and propranolol could not block the antidepressant effects of CST-14, indicating that the role of CST-14 in depression processing is not related to α/β-adrenergic receptor and CRF1 receptor. Meanwhile, Intracellular signal transduction systems have been discussed to mediate antidepressant effects such as ERK/mTOR and PI3K/Akt/mTOR pathway. The effects of some pathway inhibitiors, such as U0126 (the p-ERK inhibitor), LY294002 (the upstream Akt activator PI3K inhibitor) and rapamycin (the p-mTOR inhibitor), in the antidepressant-like effect of CST-14 revealed that there are no significant differences for U0126, LY294002 and rapamycin, showing that the antidepressant-like effect of CST-14 was not implicated in ERK/mTOR and PI3K/Akt/mTOR signal pathway.

Taken together, these findings suggested a generalized role for CST-14 in the mediation of antidepressant-related behaviors. However, studies from our laboratory also suggested that central CST-14 resulted in impairment of long-term and short-term recognition memory. Dutar et al. reported the memory-impairment effects of CST-14 in a passive avoidance paradigm (Dutar et al., 2002). Faced with these facts, no antidepressant drugs have been introduced to treat depression that act on the basis of impairing memory. For example, scopolamine, induced rapid and sustained antidepressant effects in clinical studies, but could not become such a drug (Podkowa et al., 2016). The considerable adverse effects of it, including memory impairment, hamper its use as a psychiatric drug. Thus, we propose to overcome these limitations via enhancement of the therapeutic effects of CST-14 by combining its administration with NPS, which has been widely studied as a potential anxiolytic and memory-enhancing neuropeptide, but not effect in depression in rodents (Han et al., 2013). Importantly, our data found that the joint administration of CST-14 (5 μg) and NPS (1 nmol) induced antidepressant-like effects, and did not impair cognitive function using the FST and the NOR in mice.

A great deal of evidences revealed that cyclic peptides showed enhanced tissue uptake kinetics relative to their linear and more flexible counterpart (Lättig-Tünnemann et al., 2011; Nischan et al., 2015). CST-14 consists of 14 amino acids and the sequence is P-cyclic[CKNFFWKTFSSC]-K. Thus, we evaluated intranasal administration of CST-14 on the antidepressant activity. The results showed that intranasal administration of CST-14 significantly shortened the immobility time in the FST. To demonstrate the delivery of CST-14 into the mouse brain, *ex vivo* fluorescence imaging experiments was performed. A strong NIR fluorescent signal coming from Cy7.5-CST-14 was observed over the whole brain at 30 min after intranasal injected peptide. Brains from control mice treated with Cy7.5 or saline alone displayed no significant signal. Intranasal CST-14 evokes antidepressant behavioral and this way of administration will be the potential for non-invasive depression therapeutic intervention.

## Conclusion

The present study demonstrated that central injection of CST-14 produces rapid antidepressant effects through the activation of the ghrelin receptor, GABA_A_ receptor, but not α/β-adrenergic receptor and CRF1 receptor. Meanwhile, antidepressant-like behavior of CST-14 was not related to the ERK/mTOR and PI3K/Akt/mTOR pathway. In additional, central joint administration of CST-14 and NPS induced antidepressant-like effects, and did not impair cognitive function. Finally, we find out that intranasal administration of CST-14 significantly evoked the antidepressant effects in the FST. And near-infrared fluorescent experiments were applied to detect the brain uptake and distribution of intranasal CST-14. Taken all together, the present results suggest that CST-14 might be an effective, safe and potential target for treatment of depression.

## Author Contributions

JJ and XL conducted the experiments, performed the analysis, and wrote the manuscript. YP analyzed the data and imaging. LL and XC facilitated the equipment and software to perform the behavior experiments. SL provided peptide drug CST-14. MC designed the experiments and contributed to writing and editing the manuscript. All authors read and approved the final manuscript.

## Conflict of Interest Statement

The authors declare that the research was conducted in the absence of any commercial or financial relationships that could be construed as a potential conflict of interest.
